# Medication adherence in epilepsy: Validation of the short medication adherence scale (SMAS-7) and mediation analysis of quality-of-life pathways

**DOI:** 10.1016/j.rcsop.2026.100831

**Published:** 2026-08-14

**Authors:** Mariam Dabbous, Fouad Sakr, Pascale Salameh, Pierre-Marie Preux

**Affiliations:** a Inserm U1094, IRD UMR270, Univ. Limoges, CHU Limoges, EpiMaCT - Epidemiology of chronic diseases in tropical zone, Institute of Epidemiology and Global Health – Michel Dumas, OmegaHealth, Limoges, France; b School of Pharmacy, Lebanese International University, Beirut, Lebanon; c INSPECT-LB: Institut National de Santé Publique, d'Épidémiologie Clinique et de Toxicologie-Liban, Beirut, Lebanon; d Faculty of Pharmacy, Lebanese University, Beirut, Lebanon; e Gilbert and Rose-Marie Chagoury School of Medicine, Lebanese American University, Byblos, Lebanon; f Department of Primary Care and Population Health, University of Nicosia Medical School, Nicosia, Cyprus

**Keywords:** Epilepsy, Medication adherence, Quality of life, Mediation analysis, Psychometric validation

## Abstract

**Background:**

Medication adherence is a key modifiable determinant of outcomes in epilepsy and may represent an important pathway linking psychosocial and treatment-related burdens to quality of life (QOL). However, its role within these pathways remains insufficiently understood, partly due to the lack of brief, multidimensional, validated measures in this population.

**Objectives:**

This study aimed to validate the Short Medication Adherence Scale (SMAS-7) in adults with epilepsy and to examine whether medication adherence mediates the associations of financial wellbeing, mental health, cognitive difficulties, and antiepileptic drug (AED) adverse effects with QOL.

**Methods:**

A cross-sectional study was conducted among 649 adults with epilepsy recruited from community pharmacies and a primary healthcare center. Confirmatory factor analysis, measurement invariance, reliability, construct validity, and receiver operating characteristic (ROC) analyses were used to validate the SMAS-7. Mediation analyses were performed using structural equation modeling with 5000 bootstrap resamples, adjusting for age, gender, access to healthcare, seizure characteristics, and seizure control. A Monte Carlo simulation confirmed adequate statistical power to detect indirect effects.

**Results:**

The SMAS-7 demonstrated excellent structural validity, confirming a three-factor model with excellent fit (χ^2^/df = 1.437; CFI = 0.999; TLI = 0.998; RMSEA = 0.026; SRMR = 0.015), and showed measurement invariance across gender, seizure type, and seizure control. Internal consistency was high (Cronbach's α = 0.940; McDonald's ω = 0.962). Convergent validity was excellent, and concordance with the parent instrument (LMAS-14) was very high, while divergent and concurrent validity were supported. Medication adherence partially mediated the associations of financial wellbeing (B = 0.028; 95% CI: 0.013–0.046), mental health (B = −0.037; 95% CI: −0.062 to −0.016), and AED adverse effects (B = −0.036; 95% CI: −0.058 to −0.017) with QOL, whereas the indirect effect for cognitive difficulties was not statistically significant. In a mutually adjusted sensitivity model including all four predictors simultaneously, the association between medication adherence and QOL was no longer statistically significant, and all four indirect effects were attenuated to near zero.

**Conclusion:**

The SMAS-7 is a valid, reliable, and clinically useful tool for assessing medication adherence in adults with epilepsy. When each predictor was considered individually, medication adherence showed partial indirect associations linking financial wellbeing, mental health burden, and AED adverse effects with QOL; however, these associations were attenuated to near zero in a mutually adjusted model including all four predictors simultaneously, indicating that they reflect pathway-specific associations rather than an independent mediating effect of adherence. Medication adherence nonetheless remains a clinically relevant, modifiable target within a multidimensional approach to patient-centered epilepsy care.

## Highlights

• SMAS-7 shows excellent validity, reliability, and invariance in adults with epilepsy.
• SMAS-7 briefly assesses psychological, economic, and behavioral adherence barriers.
• Adherence partially mediated financial, mental health, and AED effects on QOL.
• These associations were not retained after mutual adjustment for all predictors.
• Adherence assessment may help identify targets for improving QOL in epilepsy care.

## 1 Introduction

Epilepsy is one of the most common serious neurological disorders worldwide and is associated with substantial clinical, psychological, social, and economic burden.^1^, ^2^ Contemporary epidemiological reviews emphasize that its impact extends far beyond recurrent seizures, affecting daily functioning, participation, and long-term wellbeing across the lifespan.^1^, 3. In this context, patient-centered outcomes such as quality of life (QOL) have become essential for evaluating the overall burden of epilepsy and the real-world effectiveness of treatment.3., 4.

QOL in epilepsy is shaped by a wide range of interacting determinants. Psychiatric symptoms, particularly depression and anxiety, are among the strongest correlates of poor QOL, sometimes outweighing traditional disease markers such as seizure frequency.5., 6 This is especially important because mental health impairment is common, frequently underrecognized, and closely linked to poorer functioning and greater illness burden.^6^, ^7^ More broadly, epilepsy is increasingly recognized as a disorder accompanied by important neuropsychological and psychiatric comorbidities that must be considered alongside seizure control when assessing patient outcomes.^7^, ^8^

Cognitive difficulties and treatment-related adverse effects also contribute significantly to QOL impairment. Cognitive comorbidities may affect attention, memory, executive functioning, and daily performance, negatively influencing perceived health status.^8^, ^9^ At the same time, adverse effects of antiseizure medications remain a major determinant of reduced QOL and treatment dissatisfaction, and systematic identification of these adverse effects has been shown to improve subjective health status, underscoring their clinical relevance.^6^ Socioeconomic pressures also play an important role, as epilepsy is associated with substantial personal and financial burden.^10^ Although these determinants are well established, they are often examined as isolated correlates rather than as components of interconnected pathways influencing patient outcomes. Understanding the processes through which these determinants influence QOL may help identify modifiable targets for intervention, with medication adherence representing one plausible pathway.

One potential pathway linking these clinical and psychosocial burdens to QOL is medication adherence. Nonadherence to antiseizure medication is common and has been associated with increased seizure risk, greater healthcare utilization, and higher costs.11., 12. Adherence behavior is multifactorial, influenced by emotional distress, cognitive impairment, treatment burden, and patient-related perceptions.11., 12. Although these factors are well-established determinants of QOL, their potential indirect effects through medication adherence have received less attention despite adherence representing a modifiable target for intervention.11., 13.

An additional limitation concerns the measurement of medication adherence. Several self-report instruments, most notably the Morisky Medication Adherence Scale (MMAS-8), have been used to assess medication adherence in people with epilepsy. However, these instruments primarily provide an overall measure of adherence and were not specifically designed to capture the multidimensional psychological, economic, and behavioral factors that may underlie medication-taking behavior.11., 14 A brief instrument capturing psychological, economic, and behavioral dimensions may therefore be more practical. The Short Medication Adherence Scale (SMAS-7) was recently developed as a concise multidimensional measure.^15^ However, its factorial structure, measurement invariance, and concordance with related instruments have not been established in people with epilepsy, a population with unique cognitive, psychosocial, and pharmacological characteristics that may influence adherence patterns.12., 16 Importantly, establishing the psychometric validity of the SMAS-7 strengthens the credibility and interpretability of subsequent analyses examining medication adherence as a mediator of quality-of-life pathways.

To our knowledge, no prior study has simultaneously validated a brief adherence scale in epilepsy and examined its indirect associations with QOL pathways. Accordingly, this study had two complementary objectives: (1) to evaluate the psychometric properties of the SMAS-7 in adults with epilepsy, and (2) to investigate whether medication adherence mediates the associations between financial wellbeing, mental health, cognitive difficulties, AED adverse effects, and QOL. We hypothesized that these determinants would be associated with QOL both directly and indirectly through medication adherence.

## 2 Methods

### 2.1 Study design and participants

A cross-sectional design was employed to investigate 649 adults diagnosed with epilepsy in Lebanon. This study was reported in accordance with the Strengthening the Reporting of Observational Studies in Epidemiology (STROBE) guidelines for cross-sectional studies. Recruitment was conducted through community pharmacies across all major regions (Beirut, Mount Lebanon, North, South, and Bekaa), as well as a primary healthcare center in Beirut providing specialized epilepsy care. Participants were identified from pharmacy records and by approaching individuals seeking AED refills, either directly or via caregivers.

Eligible participants were Lebanese adults aged ≥18 years with a confirmed diagnosis of epilepsy and current use of at least one AED. Exclusion criteria included non-Lebanese individuals, age < 18 years, absence of antiepileptic treatment, and use of AEDs for non-epileptic indications (e.g., migraine prevention or weight control). All questionnaires were administered directly to the participants during face-to-face interviews. Proxy responses from caregivers or family members were not accepted, and individuals unable to provide reliable responses were not enrolled. Because recruitment was conducted consecutively across multiple community pharmacies and a primary healthcare center, the total number of eligible individuals approached was not systematically recorded; therefore, the response rate could not be calculated.

Data were collected using a structured electronic questionnaire administered through face-to-face interviews by trained interviewers. Each interview lasted approximately 20 min and was preceded by a brief explanation of the study objectives. Clinical data were verified with the treating neurologist or primary healthcare provider when necessary. The questionnaire was pilot-tested on 20 participants to assess clarity and comprehension; minor modifications were introduced, and pilot responses were excluded from the final analysis. Data collection was conducted between February and August 2025 using an Arabic version adapted to the Lebanese context.

### 2.2 Measures and variables

Data were obtained using a structured questionnaire comprising five sections. Sociodemographic characteristics (age, gender, and access to healthcare) were collected, and household crowding was calculated using the House Crowding Index (number of household members divided by the number of rooms). Financial wellbeing was assessed using the original 8-item InCharge Financial Distress/Financial Wellbeing Scale (IFDFW), a validated instrument scored from 1 to 10, with higher scores indicating better financial status.^17^ Internal consistency was high (Cronbach's α = 0.926; McDonald's ω = 0.948).

Clinical and treatment-related information included seizure characteristics and control, with verification from treating neurologists or primary care providers when required. Medication adherence was assessed using the SMAS-7, rated on a 4-point Likert scale (1 = lower adherence to 4 = higher adherence), where higher scores reflect better adherence.^15^ The Lebanese Medication Adherence Scale (LMAS-14), the parent instrument of the SMAS-7, was also administered as a reference measure. This 14-item scale, validated in neurological^18^ and cardiovascular conditions,^19^ uses a 4-point Likert format with higher scores indicating better adherence. In the present study, LMAS-14 demonstrated excellent reliability (Cronbach's α = 0.964; McDonald's ω = 0.980). As the parent instrument from which six of the seven SMAS-7 items were derived, the LMAS-14 was included to evaluate concordance between the two measures rather than as an independent validation criterion. Adverse effects related to antiepileptic treatment were measured using the Liverpool Adverse Events Profile (LAEP), a 19-item scale scored from 1 (never) to 4 (always), with higher total scores indicating greater adverse effect burden^20^, ^21^ (Cronbach's α = 0.928; McDonald's ω = 0.939).

QOL was assessed using the 15-item Quality of Life in Epilepsy Scale (QOLIE-15), which covers cognitive functioning, psychological wellbeing, therapeutic effects, seizure-related worry, and social functioning.^22^ Higher scores indicate better epilepsy-related QOL. Reliability in this sample was high (Cronbach's α = 0.906; McDonald's ω = 0.929).

Cognitive difficulties were evaluated using the A-B Neuropsychological Assessment Schedule (ABNAS), a 24-item self-report instrument assessing cognitive problems related to epilepsy and its treatment.^23^ Items are rated from 0 (no difficulty) to 3 (severe difficulty), with higher scores reflecting greater impairment (Cronbach's α = 0.976; McDonald's ω = 0.980).

Mental health was assessed using the Mental Health Assessment in Epilepsy Scale (MHAIE-11), an 11-item instrument covering three domains.^24^ Anxious-somatic distress includes five items rated from 0 (none) to 4 (very severe), depressive symptoms include four items rated from 0 (not at all) to 3 (nearly every day), and sleep sufficiency/restoration includes two items rated from 1 (always) to 5 (never). Total scores are obtained by summing all items, with higher scores indicating better mental health. Reliability was good (Cronbach's α = 0.869; McDonald's ω = 0.925). The MHAIE-11 is a newly developed measure; its factorial validity, measurement invariance, and criterion validity are reported in a dedicated psychometric validation study,^24^ and only its internal consistency in the present sample is reported here.

### 2.3 Translation procedure

Instruments were translated into Arabic following a forward-backward translation approach. The initial Arabic version was developed by a bilingual Lebanese epidemiologist. It was then independently back-translated into English by a second bilingual translator. The original and back-translated English versions were compared to ensure linguistic accuracy and cultural appropriateness, and any discrepancies were resolved through consensus.

### 2.4 Ethical aspects

The study protocol received approval from the Ethics and Research Committee of the School of Pharmacy at Lebanese International University (Approval No. 2025ERC-011-LIUSOP). Written informed consent was obtained from all participants before participation. The study was conducted in accordance with the Declaration of Helsinki, and strict measures were implemented to ensure participant confidentiality throughout.

### 2.5 Data analysis

All analyses were conducted using R version 4.5.2 (R Foundation for Statistical Computing, Vienna, Austria) within RStudio version 2026.01.0 + 392. A minimum sample size for SMAS-7 validation was determined using a participant-to-item ratio of 10:1,^25^ corresponding to at least 70 participants for the 7-item scale. The factorial structure of the SMAS-7 was examined using confirmatory factor analysis (CFA) in the *lavaan* package, based on the previously validated model in the general population.^15^ Because the SMAS-7 items are measured on a four-point ordinal Likert scale, the robust weighted least squares estimator with mean- and variance-adjusted test statistics (WLSMV) was used. Model fit was evaluated using the chi-square statistic divided by the degrees of freedom (χ^2^/df), Comparative Fit Index (CFI), Tucker–Lewis Index (TLI), Root Mean Square Error of Approximation (RMSEA), and Standardized Root Mean Square Residual (SRMR). Acceptable model fit was defined as χ^2^/df < 3, CFI and TLI ≥ 0.90, RMSEA ≤0.08, and SRMR ≤0.08.^26^, ^27^

Measurement invariance was examined using multi-group CFA across gender, seizure type (generalized vs. focal), and seizure control status (controlled vs. uncontrolled). Configural, metric, and scalar invariance were evaluated, with support established when changes in fit indices met recommended thresholds (ΔCFI ≤0.010, ΔRMSEA ≤0.015, ΔSRMR ≤0.030).^28^, ^29^

Reliability was assessed using Pearson correlations (r) among SMAS-7 items, subscales, and total score, along with polychoric-based internal consistency indices (Cronbach's α and McDonald's ω) computed using the *psych* and *semTools* packages. Construct validity was examined through correlation analyses reflecting convergent validity (LMAS-14), divergent validity (House Crowding Index), and concurrent validity (QOLIE-15, IFDFW, MHAIE-11, LAEP, and ABNAS).

Concordance with the parent instrument was evaluated using receiver operating characteristic (ROC) curve analysis implemented in the *pROC* and *ggplot2* packages. The established LMAS-14 cutoff score of 38, previously used to distinguish between higher and lower medication adherence,^19^ was used as the reference classification. The optimal SMAS-7 threshold was identified using Youden's J index, and corresponding sensitivity and specificity were reported.

Mediation analysis was conducted using the *lavaan* package within a structural equation modeling (SEM) framework with observed variables. IFDFW, MHAIE-11, ABNAS, and LAEP scores were specified as independent variables, SMAS-7 as the mediator, and QOLIE-15 as the dependent variable in four separate models adjusted for age, gender, access to healthcare, seizure characteristics, and seizure control, selected a priori based on their theoretical potential as confounders of medication adherence and QOL. Separate mediation models were specified for each predictor because the objective was to evaluate the association of medication adherence within each theoretically defined pathway rather than to estimate the independent effects of multiple correlated determinants in a single model. All models were adjusted for the same prespecified demographic and clinical covariates to improve comparability while avoiding overadjustment among conceptually related predictors. Standardized direct, indirect, and total effects, together with the proportion of the total effect mediated by adherence, were additionally computed for each model. Pearson correlations among the four predictors were also examined, and, as a sensitivity analysis, a mutually adjusted model including all four predictors simultaneously was fitted using the same covariate set and bootstrap procedure, to evaluate whether the indirect associations identified in the primary models persisted after accounting for the other psychosocial and treatment-related factors. Direct, indirect, and total effects were estimated using maximum likelihood estimation, with indirect effects evaluated via bootstrap resampling (5000 iterations) to derive bias-corrected 95% confidence intervals. Path (a) represents the effect of the independent variable on the mediator, (b) the effect of the mediator on the dependent variable, (c) the total effect of the independent variable on the dependent variable, and (c') the direct effect. Mediation was considered significant when the bootstrap confidence interval did not include zero. Results were reported as unstandardized regression coefficients (B) with standard errors (SE) and 95% confidence intervals (95% CI), while bootstrap standard errors (BootSE) and corresponding 95% confidence intervals were used for indirect effects.

A Monte Carlo simulation with 500 replications was conducted using the *lavaan* package to estimate the sample size required to detect the indirect effect in the mediation models.^30^ Based on anticipated small-to-medium effect sizes for paths (a) and (b) (*β*_*a*_ = 0.15–0.30, *β*_*b*_ = 0.30), the minimum sample size required to achieve 80% statistical power was approximately 400, based on the weakest mediation scenario. The available sample of 649 people with epilepsy provided high statistical power (94.8%–100%) across models.

## 3 Results

### 3.1 Sociodemographic and clinical characteristics

Table 1 presents the sociodemographic and clinical characteristics of the study sample. A total of 649 people with epilepsy were included, with a mean age of 34.84 years (±15.42) and a slight predominance of females (53.16%). Most participants reported easy access to healthcare (77.04%), and generalized seizures were more frequent (69.50%). Approximately two-thirds of the sample had controlled seizures (67.95%), and the mean number of antiepileptic drugs used was 1.61 (±0.82). The mean scores of the study scales were 41.14 (±16.93) for IFDFW, 42.44 (±12.47) for LAEP, 24.75 (±4.58) for SMAS-7, 45.91 (±11.28) for QOLIE-15, 25.92 (±18.59) for ABNAS, and 18.35 (±8.13) for MHAIE-11.

**Table 1:** Sociodemographic and clinical characteristics of the study sample.

Variable	Mean or Frequency	SD or %
*Age*	34.84	15.42
*Gender*		
Male	304	46.84
Female	345	53.16
*House Crowding Index*	1.19	0.54
*Easy access to healthcare*		
No	149	22.96
Yes	500	77.04
*IFDFW score*	41.14	16.93
*Seizure characteristics*		
Focal	183	28.20
Generalized	451	69.50
Unknown	15	2.30
*Level of seizure control*		
Controlled (no seizures in the last 12 months)	441	67.95
Uncontrolled (persistence of seizures despite treatment)	208	32.05
*Number of antiepileptic drugs used*	1.61	0.82
*LAEP score*	42.44	12.47
*SMAS-7 score*	24.75	4.58
*LMAS-14 score*	49.02	8.85
*QOLIE-15 score*	45.91	11.28
*ABNAS score*	25.92	18.59
*MHAIE-11 score*	18.35	8.13

SD: standard deviation; IFDFW: InCharge Financial Distress/Financial Well-Being Scale; LAEP: Liverpool Adverse Events Profile; SMAS-7: Short Medication Adherence Scale; LMAS-14: Lebanese Medication Adherence Scale; QOLIE-15: 15-item Quality of Life in Epilepsy Scale; ABNAS: A-B Neuropsychological Assessment Schedule; MHAIE-11: Mental Health Assessment in Epilepsy Scale.

### 3.2 Validation of the SMAS-7

#### 3.2.1 Confirmatory factor analysis

Table 2 presents the standardized factor loadings, standard errors, 95% confidence intervals, average variance extracted (AVE), and composite reliability (CR) for the SMAS-7. The three-factor structure showed excellent model fit (χ^2^/df = 15.804/11 = 1.437, *P* = 0.149; CFI = 0.999; TLI = 0.998; RMSEA = 0.026 [90% CI: 0.001–0.052], *P* = 0.930; SRMR = 0.015). All standardized factor loadings were statistically significant (*P* < 0.001) and ranged from 0.639 to 0.946. The AVE values ranged from 0.557 to 0.891, while composite reliability values ranged from 0.712 to 0.945, indicating adequate convergent validity and internal consistency for all three factors.

**Table 2:** Standardized factor loadings, average variance extracted, and composite reliability of the SMAS-7.

Factor	Item	λ	SE	z	*P*-value	95% CI	AVE	CR
Psychological	SMAS3	0.907	0.015	60.56	<0.001	0.878–0.937	0.852	0.945
	SMAS5	0.922	0.014	68.08	<0.001	0.895–0.949		
	SMAS4	0.940	0.012	75.83	<0.001	0.916–0.964		
Economic	SMAS6	0.942	0.013	70.44	<0.001	0.916–0.968	0.891	0.942
	SMAS7	0.946	0.011	84.89	<0.001	0.924–0.968		
Behavioral	SMAS2	0.840	0.034	24.47	<0.001	0.773–0.907	0.557	0.712
	SMAS1	0.639	0.035	18.46	<0.001	0.571–0.707		

SMAS = Short Medication Adherence Scale; λ = standardized factor loading; SE = standard error of the standardized factor loading; z = Wald test statistic; 95% CI = 95% confidence interval; AVE = average variance extracted; CR = composite reliability.

#### 3.2.2 Measurement invariance

Table 3 presents the measurement invariance of the SMAS-7 across gender, seizure characteristics, and seizure control using multigroup confirmatory factor analysis. The model demonstrated good fit at the configural, metric, and scalar levels across all groups. Changes in fit indices between nested models were minimal, with ΔCFI ranging from <0.001 to 0.003, ΔRMSEA from 0.001 to 0.005, and ΔSRMR from 0.001 to 0.011, supporting measurement invariance across the examined subgroups.

**Table 3:** Measurement invariance of the SMAS-7 across gender, seizure characteristics, and seizure control.

Model	CFI	RMSEA	SRMR	Model comparison	ΔCFI	ΔRMSEA	ΔSRMR
Model 1: across gender (male vs. female)							
Configural	0.989	0.063	0.022				
Metric	0.986	0.064	0.033	Configural vs metric	0.003	0.001	0.011
Scalar	0.986	0.059	0.034	Metric vs scalar	< 0.001	0.005	0.001

Model 2: across seizure characteristics (generalized vs. focal)							
Configural	0.991	0.057	0.018				
Metric	0.991	0.053	0.023	Configural vs metric	< 0.001	0.004	0.005
Scalar	0.988	0.056	0.027	Metric vs scalar	0.003	0.003	0.004

Model 3: across seizure control (controlled vs. uncontrolled)							
Configural	0.986	0.069	0.021				
Metric	0.985	0.065	0.027	Configural vs metric	0.001	0.004	0.006
Scalar	0.985	0.060	0.028	Metric vs scalar	< 0.001	0.005	0.001

CFI: Comparative Fit Index; RMSEA: Root Mean Square Error of Approximation; SRMR: Standardized Root Mean Square Residual.

#### 3.2.3 Internal consistency

The SMAS-7 demonstrated excellent internal consistency, with a Cronbach's α of 0.940 and a McDonald's ω of 0.962. The psychological and economic subscales also showed high internal consistency (Cronbach's α = 0.945 and 0.942, respectively), while the behavioral subscale demonstrated acceptable internal consistency (Cronbach's α = 0.746). McDonald's ω for the psychological subscale was 0.945, whereas it was not computed for the economic and behavioral subscales due to the limited number of items.

**Fig. 1:**
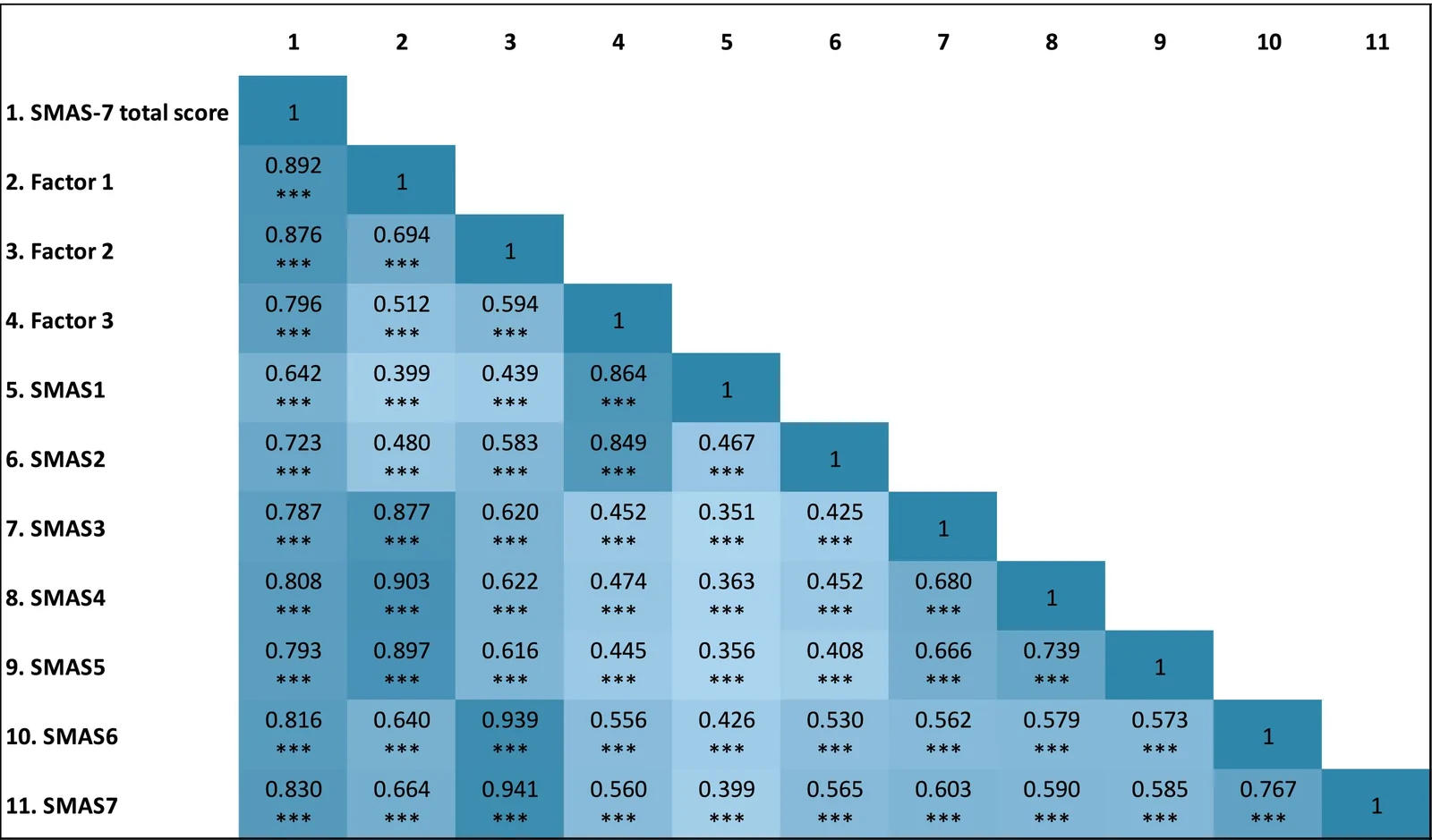
Correlation matrix of the SMAS-7 total score, factor subscales, and items. SMAS-7: 7-item Short Medication Adherence Scale; Factor 1: Psychological; Factor 2: Economic; Factor 3: Behavioral. 1: SMAS-7 total score; 2–4: Factors 1–3; 5–11: SMAS1 to SMAS7 items. ***P < 0.001.

Strong positive correlations were observed between the SMAS-7 total score and the subscales (*r* = 0.796–0.892). Correlations between items and their corresponding factors were also high, ranging from 0.849 to 0.941. Additionally, the factor subscales were significantly correlated with each other. The behavioral subscale, comprising only two items, showed comparatively weaker loadings and composite reliability than the psychological and economic subscales and should therefore be interpreted with some caution pending further evaluation in future samples. Detailed results are presented in Fig. 1.

#### 3.2.4 Convergent, divergent, and concurrent validity

The SMAS-7 demonstrated excellent convergent validity, showing a strong positive correlation with the LMAS-14 (*r* = 0.973, *P* < 0.001). Divergent validity was supported by the absence of a significant correlation with the House Crowding Index (*r* = −0.060, *P* = 0.128). Regarding concurrent validity, SMAS-7 scores were positively correlated with QOL (QOLIE-15, *r* = 0.348, *P* < 0.001) and financial wellbeing (IFDFW, *r* = 0.216, P < 0.001), and negatively correlated with mental health impairment (MHAIE-11, *r* = −0.331, *P* < 0.001), AED adverse effects (LAEP, *r* = −0.361, P < 0.001), and cognitive impairment (ABNAS, *r* = −0.360, P < 0.001).

#### 3.2.5 Concordance with the parent instrument (LMAS-14)

**Fig. 2:**
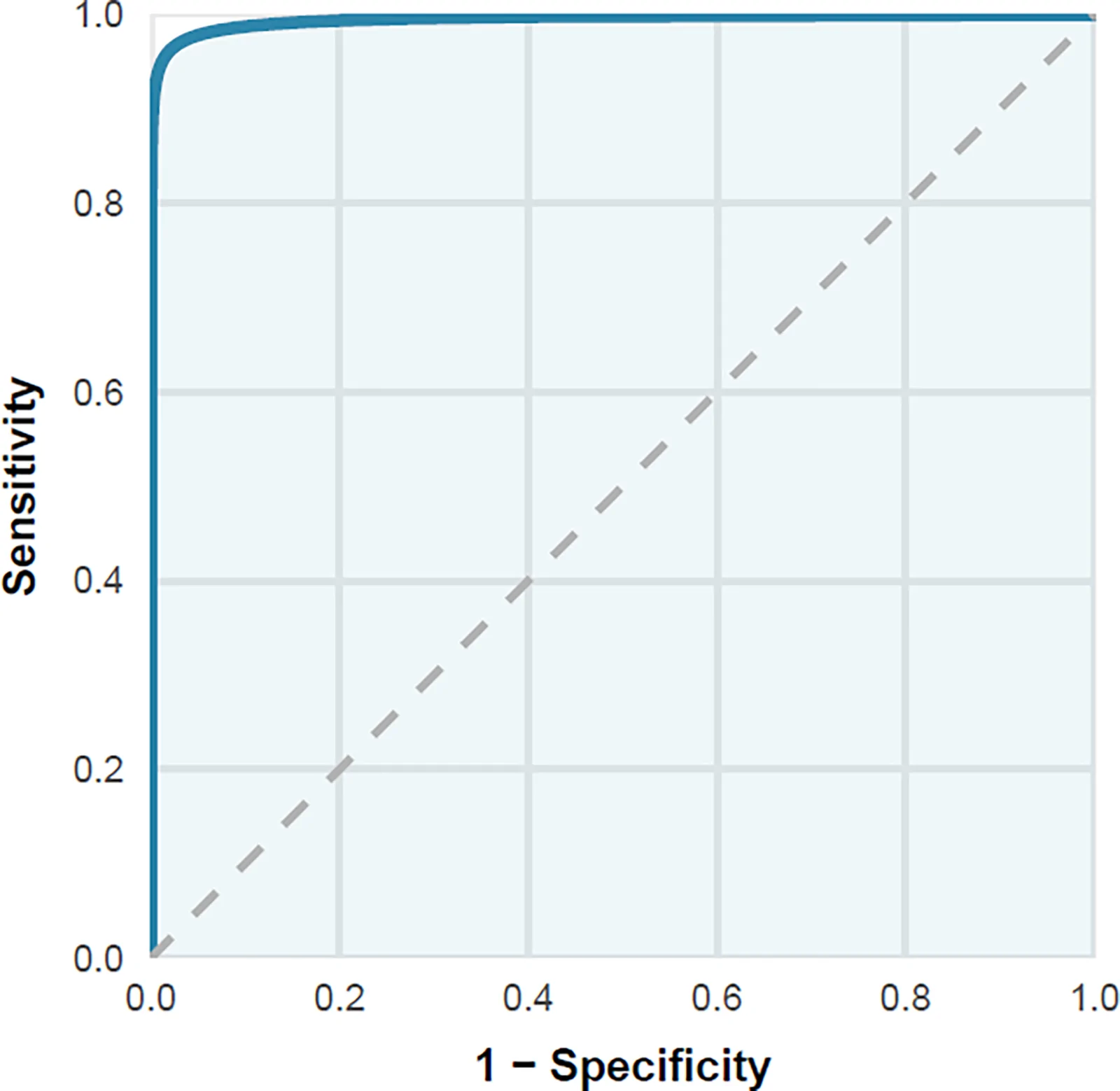
ROC curve of the SMAS-7 using the LMAS-14 cutoff point as the reference classification to classify participants with better versus lower medication adherence. Area under the curve (AUC) = 0.995 (95% CI: 0.991–0.999; *P* < 0.001). At the cutoff score of 19.5, sensitivity = 97.9% and specificity = 97.4%.

The SMAS-7 demonstrated excellent concordance with the parent instrument's classification, with an area under the curve (AUC) of 0.995 (95% CI: 0.991–0.999; P < 0.001). The optimal cutoff value based on Youden's J index was 19.5, yielding a sensitivity of 97.9% and a specificity of 97.4%. The ROC analysis, performed using the LMAS-14 cutoff point for better versus lower medication adherence as the reference classification, is presented in Fig. 2.

### 3.3 Mediation analysis

Medication adherence significantly mediated the associations between most predictors and QOL (Table 4). Indirect effects were statistically significant for financial wellbeing, mental health, and AED adverse effects, with bootstrap confidence intervals not crossing zero, indicating the presence of mediation in these models. However, the indirect effect for cognitive problems was borderline, suggesting limited evidence for mediation. As the direct effects remained statistically significant for all predictors, the mediation was partial rather than full in the significant models. The estimated indirect effects were B = 0.028 for financial wellbeing, B = −0.037 for mental health, B = −0.009 for cognitive problems, and B = −0.036 for AED adverse effects. Because these predictors were measured using different instruments and score ranges, the unstandardized indirect effects were interpreted within each predictor-specific model rather than compared across models.

**Table 4:** Adjusted mediation models examining the associations of socioeconomic, mental health, cognitive, and treatment-related factors with quality of life via medication adherence.

Predictor	Direct effect					Indirect effect				
	B	SE	95% CI		*P* value	B	BootSE	95% bootstrap CI		P Value
			Lower	Upper				Lower	Upper	
IFDFW	0.082	0.026	0.032	0.136	0.002	0.028	0.008	0.013	0.046	0.001
MHAIE-11	−1.027	0.038	−1.104	−0.954	< 0.001	−0.037	0.012	−0.062	−0.016	0.002
ABNAS	−0.470	0.015	−0.499	−0.440	< 0.001	−0.009	0.005	−0.020	0.000	0.051
LAEP	−0.505	0.029	−0.562	−0.450	< 0.001	−0.036	0.010	−0.058	−0.017	0.001

B: unstandardized regression coefficient; SE: standard error; CI: confidence interval; BootSE: bootstrap standard error; 95% bootstrap CI derived from 5000 bootstrap samples. IFDFW: InCharge Financial Distress/Financial Well-Being Scale; MHAIE-11: Mental Health Assessment in Epilepsy Scale; ABNAS: A-B Neuropsychological Assessment Schedule; LAEP: Liverpool Adverse Events Profile. All models were adjusted for age, gender, access to healthcare, seizure characteristics, and level of seizure control.

**Fig. 3:**
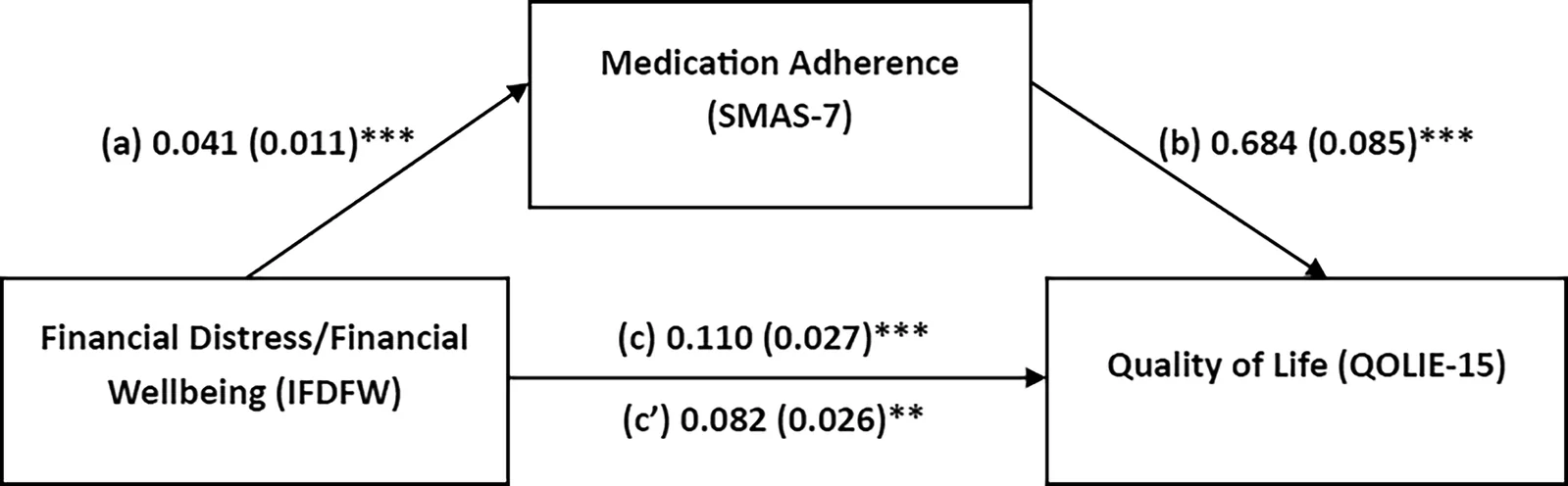
Mediation model of financial distress/financial wellbeing (IFDFW) and quality of life (QOLIE-15) via medication adherence (SMAS-7). Models were adjusted for age, gender, access to healthcare, seizure characteristics, and level of seizure control. Values are unstandardized regression coefficients (B) with (standard errors, SE). (a) effect of financial wellbeing on medication adherence; (b) effect of medication adherence on quality of life; (c) total effect of financial wellbeing on quality of life; (c') direct effect of financial wellbeing on quality of life. ***P < 0.001. **P < 0.01.

**Fig. 4:**
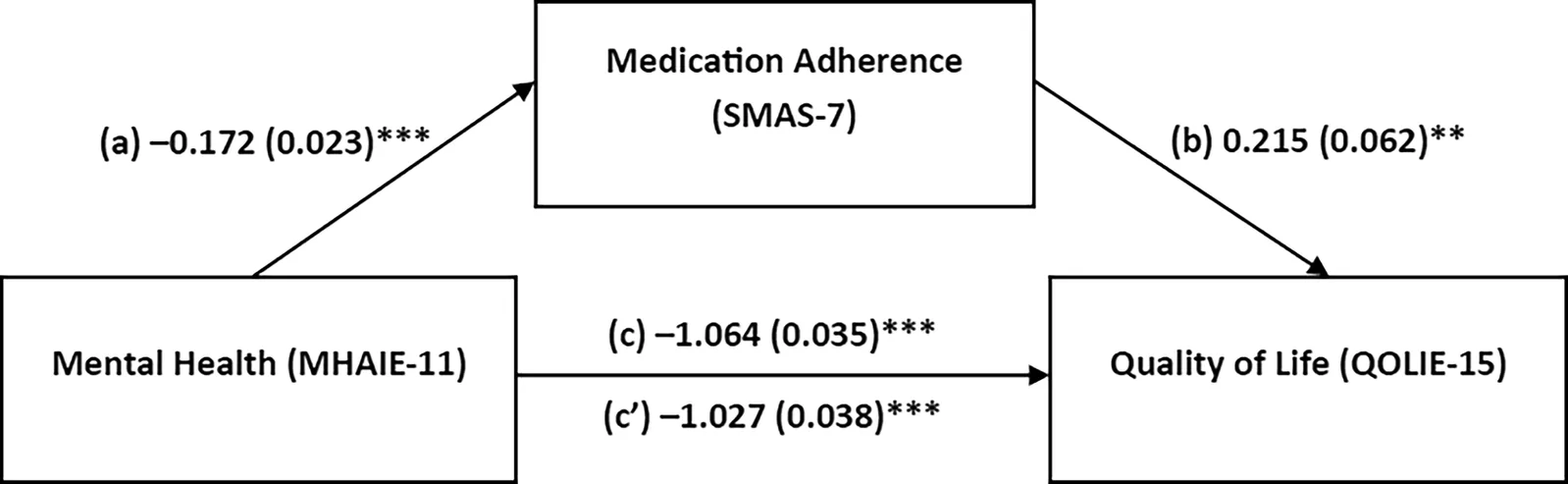
Mediation model of mental health (MHAIE-11) and quality of life (QOLIE-15) via medication adherence (SMAS-7). Models were adjusted for age, gender, access to healthcare, seizure characteristics, and level of seizure control. Values are unstandardized regression coefficients (B) with (standard errors, SE). (a) effect of mental health on medication adherence; (b) effect of medication adherence on quality of life; (c) total effect of mental health on quality of life; (c') direct effect of mental health on quality of life. ***P < 0.001. **P < 0.01.

**Fig. 5:**
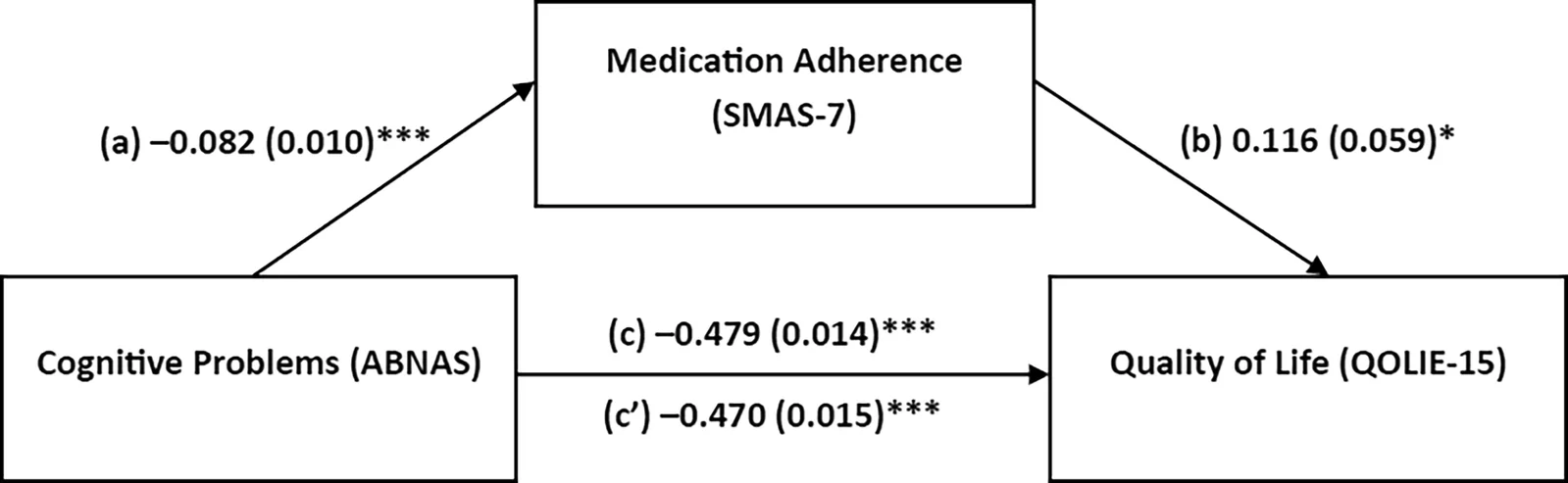
Mediation model of cognitive problems (ABNAS) and quality of life (QOLIE-15) via medication adherence (SMAS-7). Models were adjusted for age, gender, access to healthcare, seizure characteristics, and level of seizure control. Values are unstandardized regression coefficients (B) with (standard errors, SE). (a) effect of cognitive problems on medication adherence; (b) effect of medication adherence on quality of life; (c) total effect of cognitive problems on quality of life; (c') direct effect of cognitive problems on quality of life. ***P < 0.001. *P < 0.05.

**Fig. 6:**
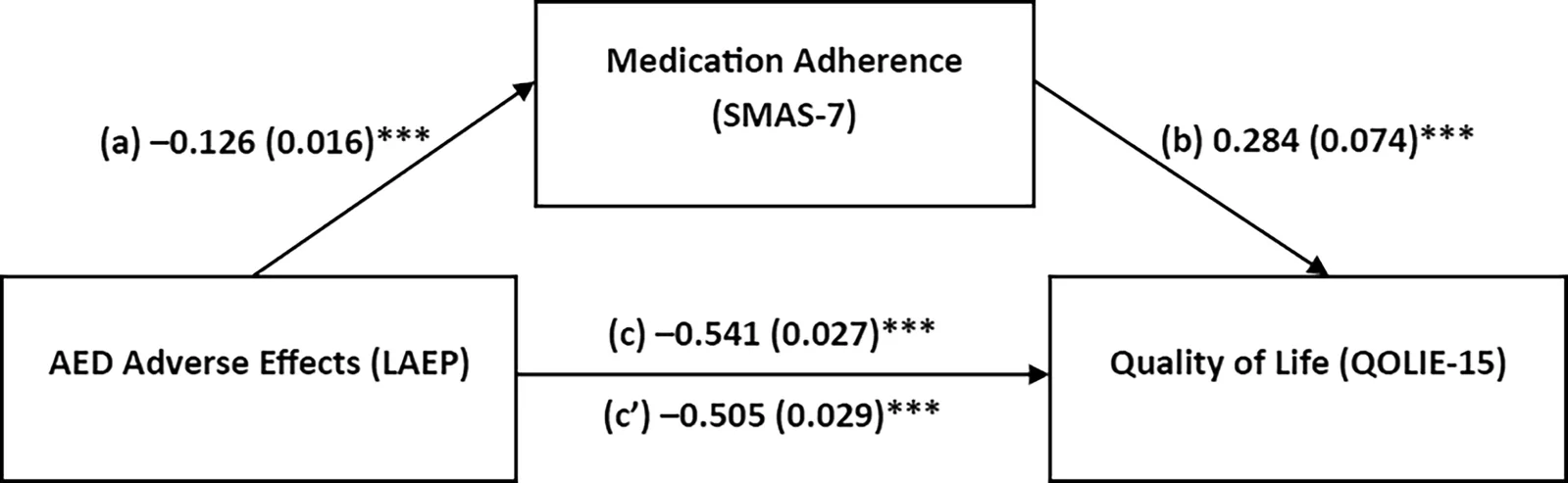
Mediation model of antiepileptic drug (AED) adverse effects (LAEP) and quality of life (QOLIE-15) via medication adherence (SMAS-7). Models were adjusted for age, gender, access to healthcare, seizure characteristics, and level of seizure control. Values are unstandardized regression coefficients (B) with (standard errors, SE). (a) effect of AED adverse effects on medication adherence; (b) effect of medication adherence on quality of life; (c) total effect of AED adverse effects on quality of life; (c') direct effect of AED adverse effects on quality of life. ***P < 0.001.

Financial wellbeing was positively associated with medication adherence and, in turn, with QOL (Fig. 3), whereas mental health burden (Fig. 4), cognitive problems (Fig. 5), and AED adverse effects (Fig. 6) were negatively associated with medication adherence and QOL. Across all models, medication adherence was consistently positively associated with QOL, further supporting its role as a mediator.

#### 3.3.1 Sensitivity analyses: predictor correlations, standardized effects, and a mutually adjusted model

Notably, the unstandardized adherence-to-QOL coefficient (path b) differed substantially across the four single-predictor models, ranging from B = 0.116 (cognitive difficulties model) to B = 0.684 (financial wellbeing model; Table 4), despite representing, in principle, the same association estimated in the same sample. Such variation is expected when a mediator's association with the outcome is estimated in models that omit correlated covariates, because unmodeled shared variance can be differentially absorbed depending on which predictor is included; this motivated the additional analyses reported below.

The four predictors were significantly intercorrelated (Table 5), with the strongest associations observed between mental health burden and cognitive difficulties (*r* = 0.696, *P* < 0.001) and between cognitive difficulties and AED adverse effects (*r* = 0.607, P < 0.001), consistent with their shared conceptual basis as overlapping facets of overall illness burden.

**Table 5:** Pearson correlations among the four predictors entered in the mediation models.

	IFDFW	MHAIE-11	ABNAS	LAEP
IFDFW	–	−0.239***	−0.242***	−0.225***
MHAIE-11	−0.239***	–	0.696***	0.606***
ABNAS	−0.242***	0.696***	–	0.607***
LAEP	−0.225***	0.606***	0.607***	–

IFDFW: InCharge Financial Distress/Financial Well-Being Scale; MHAIE-11: Mental Health Assessment in Epilepsy Scale; ABNAS: A-B Neuropsychological Assessment Schedule; LAEP: Liverpool Adverse Events Profile. ***P < 0.001.

Because the predictors were measured on different scales, standardized direct, indirect, and total effects, together with the proportion of the total effect mediated by adherence, were computed for each of the four primary models (Table 6). The proportion mediated ranged from 2.0% for cognitive difficulties to 25.4% for financial wellbeing, indicating that, even where the indirect effect was statistically significant, medication adherence accounted for a modest share of each predictor's total association with QOL.

**Table 6:** Standardized effects and proportion mediated for the primary (single-predictor) mediation models.

Predictor	Indirect effect (β)	95% CI	Total effect (β)	95% CI	Proportion mediated (%)	95% CI
IFDFW	0.042	0.017, 0.067	0.166	0.090, 0.243	25.4	11.1, 51.9
MHAIE-11	−0.027	−0.043, −0.010	−0.764	−0.795, −0.733	3.5	1.5, 5.8
ABNAS	−0.016	−0.031, 0.000	−0.794	−0.820, −0.769	2.0	0.0, 4.0
LAEP	−0.040	−0.062, −0.017	−0.602	−0.649, −0.555	6.6	3.2, 10.6

β: fully standardized coefficient (delta-method standard error); 95% CI: 95% confidence interval (delta-method for indirect and total effects; bootstrap bias-corrected accelerated for proportion mediated, 5000 resamples, computed on the unstandardized indirect/total ratio, which is scale-invariant). IFDFW: InCharge Financial Distress/Financial Well-Being Scale; MHAIE-11: Mental Health Assessment in Epilepsy Scale; ABNAS: A-B Neuropsychological Assessment Schedule; LAEP: Liverpool Adverse Events Profile.

As a sensitivity analysis addressing the possibility that the indirect effects identified in the primary models reflect shared variance among the four correlated predictors, a mutually adjusted model was fitted in which financial wellbeing, mental health, cognitive difficulties, and AED adverse effects were entered simultaneously as predictors of both medication adherence and QOL, using the same covariate set and bootstrap procedure as the primary models (Table 7). In this model, the association between medication adherence and QOL was no longer statistically significant (B = −0.001, 95% CI: −0.096 to 0.092, β = 0.000, *P* = 0.986), and all four indirect effects were correspondingly attenuated to near zero and non-significant. Mental health (β = −0.369), cognitive difficulties (β = −0.491), and AED adverse effects (β = −0.109) retained strong, statistically significant direct associations with QOL after mutual adjustment (all *P* < 0.001), whereas the association between financial wellbeing and adherence was attenuated to borderline significance (a-path *P* = 0.053). Proportion mediated is not reported for this model because the near-null indirect and total effects for most predictors render this ratio statistically unstable. These findings indicate that the indirect associations identified in the primary, single-predictor models are not independent of the substantial shared variance among mental health, cognitive difficulties, and AED adverse effects (pairwise *r* = 0.606–0.696; Table 5), and are best interpreted as evidence for each theoretically distinct pathway considered individually, rather than as evidence that medication adherence exerts an independent mediating effect once these related burdens are modeled jointly.

**Table 7:** Sensitivity analysis: mutually adjusted mediation model with all four predictors entered simultaneously.

Predictor	Path	B	95% CI	β	*P* value
IFDFW	a (→ adherence)	0.021	−0.000, 0.042	0.079	0.053
	c' (→ QOL, direct)	0.003	−0.023, 0.029	0.004	0.839
	Indirect (→ adherence → QOL)	0.000	−0.003, 0.002	0.000	0.982
MHAIE-11	a (→ adherence)	−0.040	−0.098, 0.014	−0.070	0.159
	c' (→ QOL, direct)	−0.514	−0.592, −0.434	−0.369	< 0.001
	Indirect (→ adherence → QOL)	0.000	−0.005, 0.005	0.000	0.983
ABNAS	a (→ adherence)	−0.040	−0.068, −0.013	−0.160	0.004
	c' (→ QOL, direct)	−0.296	−0.330, −0.264	−0.491	< 0.001
	Indirect (→ adherence → QOL)	0.000	−0.004, 0.005	0.000	0.985
LAEP	a (→ adherence)	−0.072	−0.109, −0.035	−0.196	< 0.001
	c' (→ QOL, direct)	−0.098	−0.142, −0.053	−0.109	< 0.001
	Indirect (→ adherence → QOL)	0.000	−0.007, 0.007	0.000	0.986
(shared)	b (adherence → QOL)	−0.001	−0.096, 0.092	0.000	0.986

B: unstandardized regression coefficient; 95% CI: 95% bootstrap bias-corrected accelerated confidence interval (5000 resamples); β: fully standardized coefficient. Model adjusted for age, gender, access to healthcare, seizure characteristics, and level of seizure control (coefficients not shown). IFDFW: InCharge Financial Distress/Financial Well-Being Scale; MHAIE-11: Mental Health Assessment in Epilepsy Scale; ABNAS: A-B Neuropsychological Assessment Schedule; LAEP: Liverpool Adverse Events Profile.

## 4 Discussion

This study makes two complementary contributions. First, it supports the SMAS-7 as a brief and psychometrically sound instrument for assessing medication adherence in adults with epilepsy. Second, it suggests that medication adherence is statistically associated with part of the relationship between broader psychosocial and treatment-related burdens and patient wellbeing, rather than being a mere correlate of QOL, thereby extending prior work that has largely treated medication adherence as a correlate rather than as a component of QOL pathways. Because the design is cross-sectional, these findings should be interpreted as indirect statistical associations consistent with the proposed pathways rather than as evidence of causal mediation. This conceptualization aligns with the growing recognition that epilepsy outcomes extend beyond seizure control to include mental health, treatment burden, and patient-reported functioning.^31^, ^32^

The validation findings support the clinical relevance of the SMAS-7. These findings are consistent with the original validation of the SMAS-7 in the general population, while extending its applicability to adults with epilepsy, a population in whom cognitive, psychosocial, and treatment-related factors may uniquely influence medication adherence. Replication of its three-domain structure is consistent with the multifactorial nature of drug adherence in epilepsy, which is influenced by psychological, behavioral, and access-related factors rather than being a purely behavioral phenomenon.^33^, 34. Its strong internal consistency, demonstrated measurement invariance across key clinical subgroups, and coherent pattern of associations with related constructs indicate that the scale captures adherence in a manner that is both psychometrically robust and clinically meaningful. The establishment of invariance is particularly important, as it supports the comparability of scores across patients with different seizure profiles and demographic characteristics and enables valid subgroup comparisons in both clinical and research settings. This is especially relevant in epilepsy care, where brief tools are needed for routine use but must still reflect the complexity of long-term AED use.34., 35 The very high agreement with the LMAS-14 further supports its utility as an efficient alternative to longer instruments. However, this should be interpreted as concordance with a related self-report measure rather than validation against objective adherence indicators.^33^, 34.

Previous epilepsy studies have primarily used adherence instruments as outcome measures or predictors of clinical outcomes, whereas comparatively little attention has been given to their role within mediation models of patient-reported outcomes. The central finding of this study is that medication adherence partially mediated the associations of financial wellbeing, mental health burden, and AED adverse effects with QOL. This pattern is consistent with the multidimensional nature of QOL in epilepsy, which reflects the combined influence of clinical, psychological, and social factors.^31^, ^32^, ^36^ The observed pattern of partial mediation is consistent with the hypothesis that adherence may represent one of several pathways through which these determinants are associated with QOL. This is clinically plausible, as QOL is shaped by multiple simultaneous processes that extend beyond treatment-taking behavior alone.^31^, ^36^ These findings suggest that medication adherence may represent one pathway through which financial wellbeing, mental health, and AED adverse effects are associated with QOL. The persistence of significant direct effects indicates that additional pathways beyond medication adherence are also likely to contribute to these associations.

The pathway involving financial wellbeing is particularly meaningful. Socioeconomic disadvantage has been consistently associated with reduced medication adherence due to barriers such as medication cost, limited access to care, and treatment interruptions.^33^, ^37^ These constraints may directly impair QOL while also indirectly affecting it through reduced adherence. In the Lebanese context, this relationship may be amplified by the ongoing economic challenges and medication shortages, which have substantially disrupted access to chronic therapies.^38^ The persistence of a direct effect therefore may reflect the broader impact of financial strain on stress, autonomy, and social participation beyond its influence on adherence.

The mediation observed for mental health burden is also well supported by the literature. Depression and anxiety are among the strongest predictors of reduced QOL in epilepsy, often exceeding the impact of seizure-related variables.^39^, ^40^ These conditions are also associated with poorer adherence and reduced self-management, making adherence a plausible behavioral pathway linking emotional distress to patient outcomes.^40^, 41. At the same time, mental health symptoms exert substantial direct effects on QOL through mechanisms such as sleep disturbance, social withdrawal, and impaired functioning, which explains the persistence of significant direct pathways.^39^, ^40^, 41.

Similarly, the findings related to AED adverse effects are consistent with prior evidence. Treatment tolerability is a key determinant of QOL, and adverse effects may negatively impact wellbeing as much as seizure burden in some patients.^36^, ^42^ Patients experiencing significant side effects may reduce or discontinue treatment, thereby affecting adherence, while adverse effects also directly impair QOL through fatigue, cognitive difficulties, and mood disturbances.^36^, ^42^ This dual pathway reinforces the importance of proactive side-effect monitoring as a central component of epilepsy management rather than a secondary consideration.

In contrast, the indirect effect of cognitive difficulties through medication adherence was borderline (*P* = 0.051), while the direct association with QOL remained statistically significant. Accordingly, the findings provide only limited evidence that medication adherence mediates the relationship between cognitive difficulties and QOL. This finding may suggest that cognitive burden affects QOL through functional and psychosocial mechanisms rather than through medication-taking behavior. Subjective cognitive complaints in epilepsy are known to be strongly influenced by emotional distress and may not closely reflect objective cognitive impairment.43., 44 As a result, their relationship with adherence may be weaker than their direct impact on daily functioning, independence, and social participation. In addition, patients may compensate for cognitive difficulties through external supports such as reminders or caregiver assistance, which may attenuate the adherence pathway while preserving their broader impact on QOL.^45^ This pathway should therefore be interpreted with caution and warrants further investigation in longitudinal studies designed to clarify the role of medication adherence in this association.

### 4.1 Implications for practice and research

These findings have important implications for both clinical practice and research. Clinically, the SMAS-7 offers a practical tool for identifying not only whether patients are nonadherent, but also why adherence is difficult, by highlighting the underlying domains contributing to this behavior. This multidimensional perspective may facilitate more targeted interventions, such as addressing psychological distress, improving treatment tolerability, or reducing financial barriers. Evidence suggests that adherence interventions in epilepsy are most effective when they are tailored and multifaceted rather than generic.^45^

From a research perspective, the results support conceptualizing adherence as a variable statistically linking other psychosocial and treatment-related factors with QOL, rather than solely as an outcome, although cross-sectional data cannot establish the direction of these relationships. Longitudinal studies are needed to confirm the directionality of these relationships and to determine whether improvements in adherence translate into sustained gains in QOL.

The decision to retain four single-predictor models as the primary analysis was based on the conceptually distinct nature of each pathway: financial wellbeing, mental health, cognitive difficulties, and AED adverse effects arise from different mechanisms and are typically addressed through different clinical interventions. From a clinical standpoint, each model identifies an actionable target that can inform individualized care regardless of a given patient's status on the other three burdens; for example, a patient presenting primarily with financial strain may benefit from adherence-focused financial counseling irrespective of their cognitive or mental health status. However, because the four predictors were substantially intercorrelated in this sample (Table 5), we also fitted a mutually adjusted sensitivity model (Section 3.3.1) in which all four predictors were entered simultaneously. In this model, the association between adherence and QOL was no longer significant, and the indirect effects were attenuated to near zero, indicating that the mediated associations identified in the primary models are not independent of the shared variance among mental health, cognitive, and treatment-related burden. This does not invalidate the individual, pathway-specific findings, which remain informative for the reasons above, but it does temper any claim that adherence exerts an independent mediating effect once these related burdens are considered jointly. Future studies with larger, multi-site samples, or approaches such as a latent ‘burden’ factor or longitudinal designs capable of separating shared from pathway-specific variance, are needed to clarify the unique contribution of each pathway.

### 4.2 Limitations and strengths

These findings should be interpreted in light of several limitations. The cross-sectional design precludes causal inference and prevented the assessment of test-retest reliability, limiting evaluation of the temporal stability of the SMAS-7. The reliance on self-reported measures introduces the possibility of reporting bias and shared method variance. In addition, the Arabic translation relied on a single forward translator rather than multiple independent forward translations with subsequent reconciliation; although independent back-translation and consensus review were performed, this is a less rigorous approach than commonly recommended multilingual validation procedures and should be considered when interpreting the linguistic equivalence of the translated instruments. In addition, concordance with the parent instrument (LMAS-14) was assessed using a related self-report measure rather than objective adherence indicators, and because six SMAS-7 items were derived from the LMAS-14, the high agreement observed should not be interpreted as independent criterion validity or external validation. Furthermore, because the four predictors were correlated with one another, the indirect associations identified in the primary models cannot fully exclude shared or confounded variance; our sensitivity analysis (Section 3.3.1) indicates that these associations should be interpreted at the level of each individual pathway rather than as evidence of an adherence-specific effect independent of the other burdens. Additionally, the behavioral subscale of the SMAS-7, comprising only two items, showed weaker loadings and composite reliability than the psychological and economic subscales (Section 3.2.3); findings involving this factor should therefore be interpreted with caution, and this component of the SMAS-7 would benefit from further psychometric evaluation in an independent sample.

However, several methodological strengths support the robustness of the findings. The study was based on a relatively large and well-powered sample with multiregional recruitment, employed a comprehensive psychometric validation framework that included structural validity and measurement invariance, and used theory-driven, adjusted mediation models with bootstrap estimation. The integration of scale validation and mediation modeling within the same study further strengthens the credibility of the mediation findings, as adherence was rigorously established as a valid construct before being examined as a mediator.

Taken together, despite these limitations, the overall pattern of results is coherent, clinically plausible, and consistent with existing literature indicating that QOL in epilepsy is shaped by the interaction of psychological, treatment-related, and socioeconomic factors rather than by seizure control alone.

## 5 Conclusion

The SMAS-7 appears to be a valid and clinically useful tool for assessing medication adherence in adults with epilepsy. Importantly, medication adherence was statistically associated with part of the relationship between financial wellbeing, mental health burden, and AED adverse effects and QOL, indicating a meaningful, albeit partial, indirect association rather than an established causal mechanism. These findings underscore that improving QOL in epilepsy requires a multidimensional approach in which medication adherence represents a key and modifiable target, but must be addressed alongside mental health, treatment tolerability, and broader social determinants of health. A sensitivity analysis mutually adjusting for all four predictors simultaneously further indicated that these indirect associations should be interpreted at the level of each individual, theoretically distinct pathway, rather than as evidence that adherence exerts an independent mediating effect once related psychosocial and treatment-related burdens are modeled jointly.

## CRediT authorship contribution statement

**Mariam Dabbous:** Conceptualization, Project administration, Methodology, Investigation, Formal analysis, Resources, Software, Data curation, Writing – original draft, Writing – review & editing, Visualization, Validation. **Fouad Sakr:** Writing – review & editing. **Pascale Salameh:** Supervision. **Pierre-Marie Preux:** Supervision. All authors reviewed and approved the final version of the manuscript.

## Consent for publication

Not applicable.

## Ethics approval and consent to participate

The study protocol received approval from the Ethics and Research Committee of the School of Pharmacy at Lebanese International University (Approval No. 2025ERC-011-LIUSOP). Written informed consent was obtained from all participants before participation. The study was conducted in accordance with the Declaration of Helsinki, and strict measures were implemented to ensure participant confidentiality throughout.

## Funding

This research did not receive any specific grant from funding agencies in the public, commercial, or not-for-profit sectors.

## Data statement

The datasets generated during and/or analyzed during the current study are available from the corresponding author upon reasonable request.

## Declaration of competing interest

The authors have no relevant financial or non-financial interests to disclose.

## References

[1] (2024). Global, regional, and national burden of disorders affecting the nervous system, 1990-2021: a systematic analysis for the global burden of disease study 2021. Lancet Neurol.

[2] (2019). Global, regional, and national burden of neurological disorders, 1990-2016: a systematic analysis for the global burden of disease study 2016. Lancet Neurol.

[3] Devinsky O., Vezzani A., O'Brien T.J. (2018). Epilepsy. Nat Rev Dis Primers.

[4] Michaelis R., Tang V., Wagner J.L. (2017). Psychological treatments for people with epilepsy. Cochrane Database Syst Rev.

[5] Camara-Lemarroy C.R., Hoyos M., Ibarra-Yruegas B.E., Díaz-Torres M.A., De León R. (2017). Affective symptoms and determinants of health-related quality of life in Mexican people with epilepsy. Neurol Sci.

[6] Blond B.N., Detyniecki K., Hirsch L.J. (2016). Assessment of treatment side effects and quality of life in people with epilepsy. Neurol Clin.

[7] Keezer M.R., Sisodiya S.M., Sander J.W. (2016). Comorbidities of epilepsy: current concepts and future perspectives. Lancet Neurol.

[8] Brooks-Kayal A.R., Bath K.G., Berg A.T. (2013). Issues related to symptomatic and disease-modifying treatments affecting cognitive and neuropsychiatric comorbidities of epilepsy. Epilepsia.

[9] Leeman-Markowski B.A., Schachter S.C. (2016). Treatment of cognitive deficits in epilepsy. Neurol Clin.

[10] Grabowski D.C., Fishman J., Wild I., Lavin B. (2018). Changing the neurology policy landscape in the United States: misconceptions and facts about epilepsy. Health Policy.

[11] Malek N., Heath C.A., Greene J. (2017). A review of medication adherence in people with epilepsy. Acta Neurol Scand.

[12] Brodtkorb E., Samsonsen C., Sund J.K., Bråthen G., Helde G., Reimers A. (2016). Treatment non-adherence in pseudo-refractory epilepsy. Epilepsy Res.

[13] Hamedi-Shahraki S., Eshraghian M.R., Yekaninejad M.S. (2019). Health-related quality of life and medication adherence in elderly patients with epilepsy. Neurol Neurochir Pol.

[14] Turan G.B., Demir M. (2026). Factors associated with antiseizure medication adherence in patients with epilepsy: a systematic review. Brain Behav.

[15] Sakr F., Dabbous M., Safwan J., Rahal M., Salameh P. (2025). The short medication adherence scale (SMAS-7): development and psychometric validation in a general population sample. Explor Res Clin Soc Pharm.

[16] Michaelis R., Tang V., Goldstein L.H. (2018). Psychological treatments for adults and children with epilepsy: evidence-based recommendations by the international league against epilepsy psychology task force. Epilepsia.

[17] Sakr F., Dabbous M., Safwan J., El Bakri M., Rahal M., Salameh P. (2026). The 6-item InCharge financial distress/financial wellbeing (IFDFW-6) scale: development and validation in pharmacy and epidemiology research. Explor Res Clin Soc Pharm.

[18] Sakr F., Dabbous M., Akel M., Salameh P., Hosseini H. (2022). Adherence to post-stroke pharmacotherapy: scale validation and correlates among a sample of stroke survivors. Medicina (Kaunas).

[19] Bou Serhal R., Salameh P., Wakim N. (2018). A new Lebanese medication adherence scale: validation in Lebanese hypertensive adults. Int J Hypertens.

[20] Dibek D.M., Kılıç B., Oztura I., Baklan B. (2023). The Liverpool adverse drug events profile (LAEP): validity and reliability of Turkish version (LAEP-TR). Neurol Sci Neurophysiol.

[21] Sokić N., Ristić A.J., Bukumirić Z., Vojvodić N., Kovačević M., Sokić D. (2020). Validation of the Serbian version of the Liverpool adverse events profile of antiseizure therapy in patients with epilepsy. Epilepsy Behav.

[22] Dabbous M., Sakr F., Salameh P., Preux P.M. (2026). Development, psychometric validation, and correlates of the 15-item quality of life in epilepsy scale (QOLIE-15). Sci Rep.

[23] Aldenkamp A.P., van Meel H.F., Baker G.A., Brooks J., Hendriks M.P. (2002). The A-B neuropsychological assessment schedule (ABNAS): the relationship between patient-perceived drug related cognitive impairment and results of neuropsychological tests. Seizure.

[24] Dabbous M., Sakr F., Preux P.-M., Salameh P. (2026).

[25] Boateng G.O., Neilands T.B., Frongillo E.A., Melgar-Quiñonez H.R., Young S.L. (2018). Best practices for developing and validating scales for health, social, and behavioral research: a primer. Front Public Health.

[26] Hu L.t., Bentler P.M. (1999). Cutoff criteria for fit indexes in covariance structure analysis: conventional criteria versus new alternatives. Struct Equ Model Multidiscip J.

[27] Schermelleh-Engel K., Moosbrugger H., Müller H. (2003). Evaluating the fit of structural equation models: tests of significance and descriptive goodness-of-fit measures. Methods Psychol Res Online.

[28] Chen F.F. (2007). Sensitivity of goodness of fit indexes to lack of measurement invariance. Struct Equ Model Multidiscip J.

[29] Putnick D.L., Bornstein M.H. (2016). Measurement invariance conventions and reporting: the state of the art and future directions for psychological research. Dev Rev.

[30] Thoemmes F., MacKinnon D.P., Reiser M.R. (2010). Power analysis for complex mediational designs using Monte Carlo methods. Struct Equ Model Multidiscip J.

[31] Taylor R.S., Sander J.W., Taylor R.J., Baker G.A. (2011). Predictors of health-related quality of life and costs in adults with epilepsy: a systematic review. Epilepsia.

[32] Baker G.A. (2002). The psychosocial burden of epilepsy. Epilepsia.

[33] Eatock J., Baker G.A. (2007). Managing patient adherence and quality of life in epilepsy. Neuropsychiatr Dis Treat.

[34] Faught E. (2012). Adherence to antiepilepsy drug therapy. Epilepsy Behav.

[35] Stafford M., Gavriel S., Lloyd A. (2007). Patient-reported outcomes measurements in epilepsy. Expert Rev Pharmacoecon Outcomes Res.

[36] Baker G.A., Jacoby A., Buck D., Stalgis C., Monnet D. (1997). Quality of life of people with epilepsy: a European study. Epilepsia.

[37] Shallcross A.J., Becker D.A., Singh A. (2015). Psychosocial factors associated with medication adherence in ethnically and socioeconomically diverse patients with epilepsy. Epilepsy Behav.

[38] Khattar G., Hallit J., El Chamieh C., Bou Sanayeh E. (2022). Cardiovascular drug shortages in Lebanon: a broken heart. Health Econ Rev.

[39] Boylan L.S., Flint L.A., Labovitz D.L., Jackson S.C., Starner K., Devinsky O. (2004). Depression but not seizure frequency predicts quality of life in treatment-resistant epilepsy. Neurology.

[40] Beyenburg S., Mitchell A.J., Schmidt D., Elger C.E., Reuber M. (2005). Anxiety in patients with epilepsy: systematic review and suggestions for clinical management. Epilepsy Behav.

[41] Quon R., Andrew A., Schmidt S. (2019). Self-management practices associated with quality of life for adults with epilepsy. J Neurol.

[42] Yue L., Yu P.M., Zhao D.H. (2011). Determinants of quality of life in people with epilepsy and their gender differences. Epilepsy Behav.

[43] Galioto R., Blum A.S., Tremont G. (2015). Subjective cognitive complaints versus objective neuropsychological performance in older adults with epilepsy. Epilepsy Behav.

[44] Liik M., Vahter L., Gross-Paju K., Haldre S. (2009). Subjective complaints compared to the results of neuropsychological assessment in patients with epilepsy: the influence of comorbid depression. Epilepsy Res.

[45] Al-Aqeel S., Gershuni O., Al-Sabhan J., Hiligsmann M. (2020). Strategies for improving adherence to antiepileptic drug treatment in people with epilepsy. Cochrane Database Syst Rev.

